# Forkhead Domains of FOXO Transcription Factors Differ in both Overall Conformation and Dynamics

**DOI:** 10.3390/cells8090966

**Published:** 2019-08-24

**Authors:** Katarina Psenakova, Klara Kohoutova, Veronika Obsilova, Michael J. Ausserlechner, Vaclav Veverka, Tomas Obsil

**Affiliations:** 1 Department of Physical and Macromolecular Chemistry, Faculty of Science, Charles University, 128 43 Prague, Czech Republic; 2 Department of Structural Biology of Signaling Proteins, Division BIOCEV, Institute of Physiology of the Czech Academy of Sciences, 252 50 Vestec, Czech Republic; 3 Department of Pediatrics I, Medical University Innsbruck, A-6020 Innsbruck, Austria; 4 Institute of Organic Chemistry and Biochemistry of the Czech Academy of Sciences, 166 10 Prague, Czech Republic; 5 Department of Cell Biology, Faculty of Science, Charles University, 128 43 Prague, Czech Republic

**Keywords:** FOXO1, Forkhead domain, structure, DNA-binding domain, nuclear magnetic resonance

## Abstract

FOXO transcription factors regulate cellular homeostasis, longevity and response to stress. FOXO1 (also known as FKHR) is a key regulator of hepatic glucose production and lipid metabolism, and its specific inhibition may have beneficial effects on diabetic hyperglycemia by reducing hepatic glucose production. Moreover, all FOXO proteins are considered potential drug targets for drug resistance prevention in cancer therapy. However, the development of specific FOXO inhibitors requires a detailed understanding of structural differences between individual FOXO DNA-binding domains. The high-resolution structure of the DNA-binding domain of FOXO1 reported in this study and its comparison with structures of other FOXO proteins revealed differences in both their conformation and flexibility. These differences are encoded by variations in protein sequences and account for the distinct functions of FOXO proteins. In particular, the positions of the helices H1, H2 and H3, whose interface form the hydrophobic core of the Forkhead domain, and the interactions between hydrophobic residues located on the interface between the N-terminal segment, the H2-H3 loop, and the recognition helix H3 differ among apo FOXO1, FOXO3 and FOXO4 proteins. Therefore, the availability of apo structures of DNA-binding domains of all three major FOXO proteins will support the development of FOXO-type-specific inhibitors.

## 1. Introduction

The members of the Forkhead box (FOX) family of transcription factors share a conserved winged-helix DNA-binding domain (DBD) known as the Forkhead domain [1,2]. This domain consists of approximately 110 amino acids and folds into three major α-helices (H1-H3), a short twisted three-stranded antiparallel β-sheet (comprising three β strands S1–S3) and two wing-like loops (W1 and W2), and their arrangement within the Forkhead domain is H1-S1-H2-H3-S2-W1-S3-W2 [3]. The third helix H3 is the main DNA recognition element that binds to the major groove roughly perpendicularly to the DNA axis and makes most of the base-specific contacts with the core sequence [3,4,5,6,7,8]. Furthermore, the DNA-binding surface also includes the loop region W1, the loop between helices H4 and H3 and the N-terminal segment preceding the helix H1. In comparison with other FOX subclasses, the Forkhead domains of FOXO proteins contain a 5-amino-acid insertion between α-helices H2 and H3 (residues KGDSN). All FOXO-DBDs recognize consensus sequences 5′-GTAAACAAtab-3′ and 5′-(C/A)(A/C)AAA(C/T)AA-3′ known as the DAF-16 family member-binding element (DBE) and the insulin responsive element (IRE), respectively, which include the core sequence 5′-(A/C)AA(C/T)A-3′ recognized by all Forkhead transcription factors [9,10,11,12]. However, the factors that contribute to DNA binding specificity to diverse DNA sequences adjacent to the core sequence among individual FOX members are still not fully understood [13,14]. All FOXO proteins bind to DNA duplexes as monomers similarly to other Forkhead proteins [3,4,5,6,7,8].

**Figure 1:**
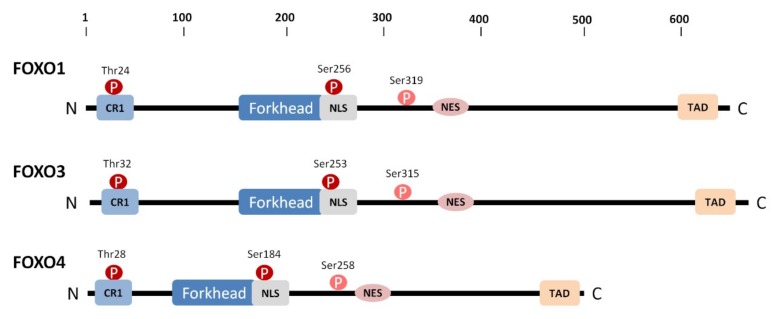
Domain structure of FOXO proteins. The positions of PKB/Akt phosphorylation sites are indicated by circles. The first two PKB/Akt phosphorylation sites are also binding motifs for the scaffolding 14-3-3 protein. CR1, conserved region 1; NLS, nuclear localization signal; NES, nuclear export signal; TAD (CR3), transactivation domain.

The “O” subclass of FOX family consists of four proteins (FOXO1/FKHR, FOXO3/FKHRL1, FOXO4/AFX and FOXO6), which regulate cellular homeostasis, longevity and response to stress by modulating diverse cellular functions, including cell cycle, stress resistance, DNA damage repair, apoptosis, tumor resistance and metabolism (reviewed in [15,16,17]). The transcriptional activity of FOXO proteins is negatively regulated by protein kinase B (PKB, also known as Akt), which phosphorylates three Ser/Thr residues and induces binding to the scaffolding protein 14-3-3. In turn, 14-3-3 protein binding sterically obscures both the nuclear localization sequence and the Forkhead domain of FOXO proteins, thereby shifting the equilibrium of FOXO localization toward the cytoplasm [18,19,20,21,22]. In addition to the Forkhead domain, FOXO proteins contain two other conserved regions (CR1 and CR3) located within long and presumably disordered segments bordering the Forkhead domain (Figure 1). CR1 is positioned at the N-terminus and contains the first PKB phosphorylation site, which is also a 14-3-3 protein-binding motif, whereas CR3 is located at the C-terminus and represents the transactivation domain [23]. The nuclear localization sequence partly overlaps with the C-terminal part of the Forkhead domain and contains the second PKB phosphorylation site/14-3-3-binding motif. The third PKB phosphorylation site, which is not the 14-3-3 binding motif, is located approximately 60 residues downstream of the second site.

FOXO proteins are further regulated through a number of additional posttranslational modifications, including phosphorylation, acetylation, ubiquitination, and methylation (reviewed in [24,25,26]). Sites of these modifications are localized within both the Forkhead domain and long flexible regions bordering the Forkhead domain, and their modifications affect the stability of FOXO proteins, binding to the target DNA, and interactions with other binding partners [18,19,27,28,29,30]. 

FOXO proteins were initially considered potent tumor suppressors due to their ability to induce cell cycle arrest and apoptosis; however, recent studies have shown that FOXO proteins can also promote tumor development and progression by maintaining cellular homeostasis and by inducing drug-resistance [31,32,33]. Moreover, the resistance to cytotoxic chemotherapeutics is linked to deregulated signaling not only through FOXO proteins but also FOXM1, which is a potent oncogene whose expression and activity are negatively regulated by FOXO3 [34,35]. FOXO3 and FOXM1 exert opposing functions in the regulation of cancer-related processes, and targeting the FOXO3-FOXM1 axis could be a viable strategy for the treatment of cancer. Therefore, blocking FOX transcriptional activities with specific inhibitors may help to prevent drug resistance in cancer therapy. Previous studies have shown that selective pharmacological inhibition of FOXO1 may have beneficial effects on diabetic hyperglycemia by reducing hepatic glucose production [36,37]. Several small-molecule compounds reported by Langlet et al. [37] were shown to specifically clear FOXO1 from glucose-6-phosphatase promoter. However, design and/or further optimization of specific FOXO inhibitors requires a detailed understanding of structural differences between individual FOXO-DBDs. Structures of both apo and DNA-bound forms of FOXO3-DBD and FOXO4-DBD and of the FOXO1-DBD:DNA complex have already been reported. In this study, we report a high-resolution solution structure of apo FOXO1-DBD, which allowed us to systematically compare all three major FOXO-DBDs in both their apo and DNA-bound forms. This comparison revealed that apo FOXO-DBDs differ in mutual positions of the helices H1, H2 and H3, that form the Forkhead domain core and in interactions between hydrophobic regions within the N-terminal segment, the H2-H3 loop, and the recognition helix H3. Moreover, FOXO proteins also show variations in conformational heterogeneity in the H4-H3 loop and in the N-terminal part of the helix H3.

## 2. Materials and Methods

### 2.1. FOXO1-DBD Expression and Purification 

DNA encoding mouse FOXO1-DBD (residues 156−269) was ligated into the pGEX-6P-1 vector (GE Healthcare Bio-Sciences, Pittsburgh, PA, USA) using the BamHI and XhoI sites. ^13^C/^15^N- and ^15^N-labeled FOXO1-DBD was expressed as an N-terminal GST-tagged fusion protein in *Escherichia coli* BL21(DE3) cells grown in minimal medium containing ^15^N-ammonium sulfate and/or ^13^C-glucose as the sole nitrogen and carbon source. Protein expression was induced by 0.5 mM isopropyl β-d-1-thiogalactopyranoside for 18 h at 20 °C. Pelleted cells were resuspended in buffer 2× PBS, 10 mM dithiothreitol (DTT), 1 mM EDTA, and lysed by sonication at 4 °C. The protein was purified using Glutathione Sepharose 4 Fast Flow (GE Healthcare Bio-Sciences, Pittsburgh, PA, USA) in buffer containing 20 mM Tris-HCl, 0.5 M NaCl, 10 mM DTT, 1 mM EDTA, 10% (*w/v*) glycerol at pH 7.5. The fusion protein was eluted at room temperature using 10 mM glutathione and dialyzed against buffer containing 20 mM Tris-HCl, 100 mM NaCl, 1 mM EDTA, 1 mM DTT, 10% (*w/v*) glycerol at pH 8.0. The affinity tag was removed by PreScission Protease cleavage overnight at 4 °C (10 U/mg recombinant fusion protein). After cleavage, FOXO1-DBD was purified by size-exclusion chromatography (HiLoad Superdex 75; GE Healthcare) in 20 mM phosphate buffer containing 50 mM KCl, 1 mM TCEP, 1 mM EDTA and 10% (*w/v*) glycerol at pH 6.5. 

### 2.2. NMR Spectroscopy 

NMR spectra were acquired at 25 °C on Bruker Avance III™ HD 600 MHz and 850 MHz spectrometers, both equipped with a ^1^H/^13^C/^15^N cryoprobe. The 350-μL sample of 380 μM ^13^C/^15^N-labeled FOXO1-DBD in buffer containing 20 mM sodium phosphate at pH 6.5, 50 mM KCl, 1 mM TCEP, 1 mM EDTA and 10% D_2_O/90% H_2_O was used for the sequence-specific backbone resonance assignment. The protein spectra were affected by C-terminal degradation, which was manifested as peak doubling and signal intensity reduction over time. Protein fitness was assessed by regular acquisition of 2D ^1^H-^15^N HSQC spectra. In total, protein samples from three independent purifications were used for NMR data collection. The spectra were processed using Bruker Topspin 3.5 and were analyzed in NMRFAM-SPARKY [38]. Sequence-specific backbone and side-chain resonance assignment was obtained using a series of standard triple-resonance spectra (HNCO, HN(CA)CO, HNCACB, CBCA(CO)NH, HBHA(CO)NH, CCC(CO)HN and HCCH-TOCSY, experiments) [39,40]. In particular, 104 of the 114 (91%) backbone amide signals in the 2D ^1^H-^15^N HSQC spectrum of FOXO1-DBD were assigned (Supplementary Figure S1). A set of ^1^H-^1^H distance constraints required for structural determination was obtained from intensities of NOE cross peaks in the 3D ^15^N/^1^H NOESY-HSQC and ^13^C/^1^H NOESY-HMQC spectra, both acquired with τm = 120 ms. This yielded unique assignments for 95.8% (3006/3139) of the NOE peaks, providing 1712 non-redundant ^1^H-^1^H distance constraints.

### 2.3. NMR Structure Calculation

FOXO1-DBD structural calculation was performed in Cyana 3.98 using the combined automated NOE assignment and structure determination protocol (Candid) [41] followed by five cycles of simulated annealing combined with redundant dihedral angle constraints (Redac) [42]. The NOESY data were complemented by the backbone torsion angle constraints obtained from the NMR resonance assignments using TALOS+ [43] as an input. The resulting set of FOXO1-DBD converged structures with no NOE-derived distance constraints and van der Waals violations greater than 0.5 Å or dihedral angle constraint violation greater than 5° were refined in explicit water using YASARA [44]. The 30 FOXO1-DBD structures with the lowest total energy were selected, analyzed and validated using the Protein Structure Validation Software suite (http://psvs-1_5-dev.nesg.org). The constraints and structural quality statistics for the final water-refined set of FOXO1-DBD structures are summarized in Table 1. The structures, NMR resonance assignments and constraints used in structural calculation were deposited in the Protein Data Bank (PDB code: 6QVW) and Biological Magnetic Resonance Bank (BMRB code: 34364).

## 3. Results and Discussion

### 3.1. Solution Structure of the DNA-binding Domain of FOXO1

**Figure 2:**
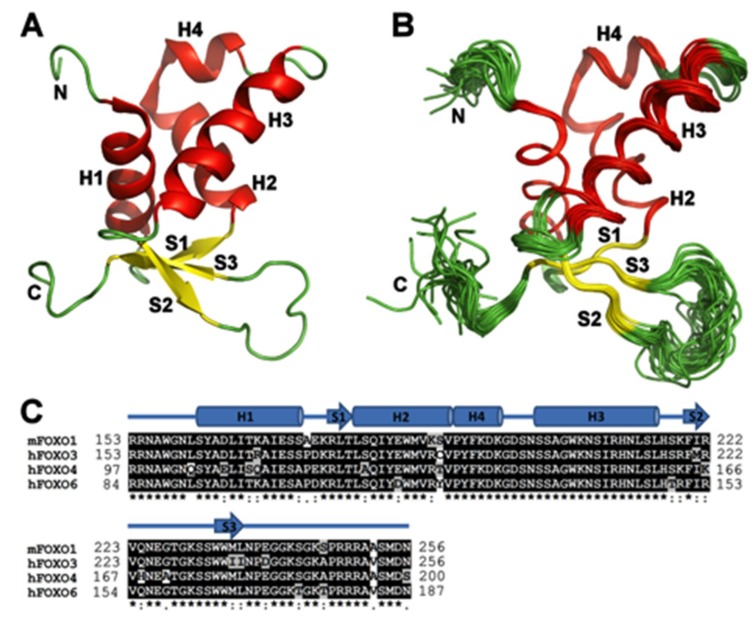
Solution structure of FOXO1-DBD. (**A**) Cartoon representation of the NMR solution structure of FOXO1-DBD showing residues 156-243. Secondary structure elements are labeled according to the nomenclature typical of the winged-helix motif [3]. (**B**) 30 superimposed FOXO1-DBD conformers of the NMR solution structure ensemble. (**C**) Sequence alignment of FOXO forkhead domains. Secondary-structure elements (based on the FOXO1-DBD) are indicated at the top. Protein sequences of mouse and human FOXO1-DBD are identical.

For detailed understanding of structural differences between individual FOXO DNA-binding domains, we determined the solution structure of the apo FOXO1-DBD. FOXO1-DBD adopts the expected Forkhead winged-helix fold (Figure 2A,B), and similar to FOXO3-DBD and FOXO4-DBD structures, the region between helices H2 and H3 of FOXO1-DBD contains an additional short helix, H4, which partly overlaps with the N-terminal end of the 5-amino-acid insertion [45,46]. A well-defined structure with an average root mean square deviation (R.M.S.D.) for C_α_ atoms less than 1.0 Å was obtained for residues of the α-helices H1, H2 and H4, β-strand S1, C-terminal half of α-helix H3, and β-strands S2 and S3 (Figure 2B). Conversely, higher conformational heterogeneity, which reflects smaller numbers of experimentally derived restrains, presumably due to increased dynamics, was observed in the N-terminal segment, N-terminal half of the α-helix H3, loop between H3 and S2 and both loops W1 and W2, which is in the regions involved in DNA binding [6,7,8].

### 3.2. Comparison of FOXO1-DBD with Forkhead Domains of Other FOXO Proteins

**Figure 3:**
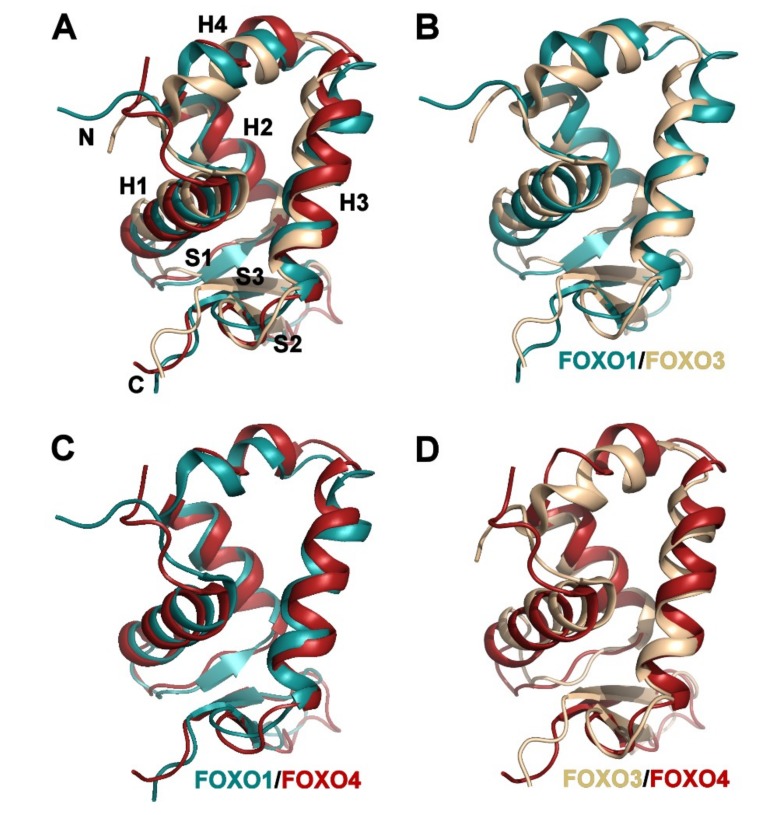
Comparison of FOXO1-DBD with previously reported solution structures of FOXO3-DBD and FOXO4-DBD. (**A**) Superimposition of representative conformers of FOXO1-DBD (conf. no. 16), FOXO3-DBD (conf. no. 11, PDB ID: 2K86 [46]) and FOXO4-DBD (conf. no. 3, PDB ID: 1E17 [45]). Selected conformers are representatives of the most populated clusters obtained by cluster analysis of conformational ensembles of NMR solution structures [47]. Structural comparison was performed using residues 156–239 (mouse FOXO1 numbering). Structures were superimposed using all C_α_ atoms. (**B**–**D**) Superimposition of FOXO1/FOXO3, FOXO1/FOXO4 and FOXO3/FOXO4 DBDs.

**Figure 4:**
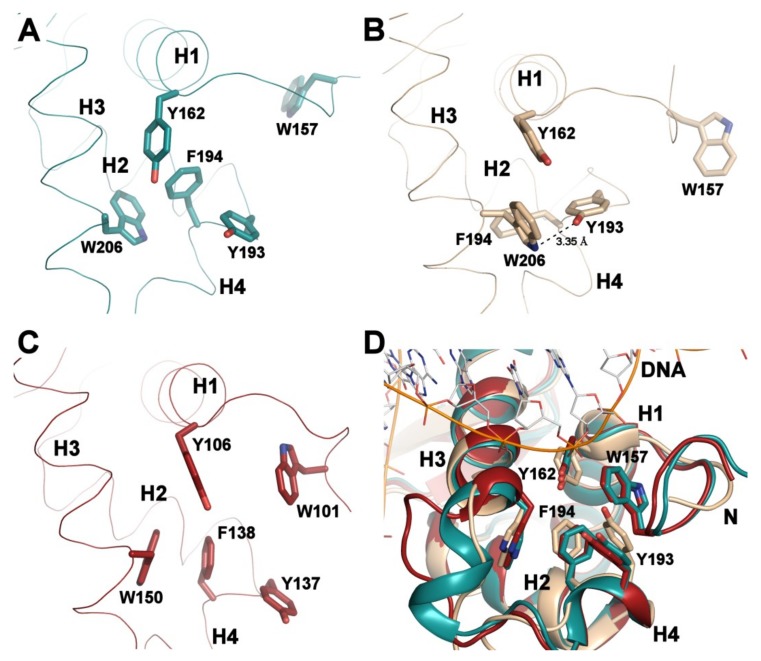
Interactions of hydrophobic residues on the interface between the N-terminal segment, the H2–H3 loop and helix H3. (**A**) Interactions observed in apo FOXO1-DBD (conf. no. 16). (**B**) Interactions observed in apo FOXO3-DBD (conf. #11, PDB ID: 2K86 [46]). (**C**) Interactions observed in apo FOXO4-DBD (conf. no. 3, PDB ID: 1E17 [45]). (**D**) Interactions observed in DNA-bound forms of FOXO1-DBD (PDB ID: 3COA [7], shown in dark cyan), FOXO3-DBD (PDB ID: 2UZK [6], shown in sand) and FOXO4-DBD (PDB ID: 3L2C [8], shown in dark red). For clarity, only DNA in the FOXO1-DBD:DNA complex is shown.

Because the previously reported solution structures of apo FOXO3-DBD (PDB ID: 2K86 [46]) and apo FOXO4-DBD (PDB ID: 1E17 [45]) were solved with constructs of different lengths, apo FOXO-DBDs were structurally compared using residues 156-239 (mouse FOXO1 numbering) present in all three structures. The superimposition of representative conformers (representatives of the most populated clusters assessed by cluster analysis of conformational ensembles of NMR solution structures [47]) revealed that the apo FOXO1-DBD can be superimposed with the solution structures of apo FOXO3-DBD and FOXO4-DBD with root-mean-square deviations (R.M.S.D.) of 2.05 and 2.12 Å, respectively, over 84 C_α_ atoms (Supplementary Table S1). FOXO3-DBD and FOXO4-DBD can be superimposed with the R.M.S.D. of 2.50 Å over 84 C_α_ atoms. The most significant differences are observed in the loop between helices H2 and H3, which contains the additional helix H4, and the N-terminal half of helix H3, whose orientation differs among all three FOXO-DBDs (Figure 3 and Figure S2). Structural superimposition and difference residue-residue distance maps calculated for selected conformers also suggest that the mutual positions of the α-helices H1, H2 and H3, whose interface form the hydrophobic core of the domain, apparently differ among apo-FOXO-DBDs. To describe these differences, three isoleucine residues located approximately in the middle of all three helices in regions with high backbone coordinate precision were selected (I166, I183 and I210 in the case of FOXO1), and the distances of their C_α_ atoms were compared (Figure S3). The results suggested that FOXO1-DBD is more compact (has shorter H2-H3 and H1-H2 distances) than the other two FOXO proteins, whereas FOXO4-DBD appears to be less compact, based on the sum of C_α_-C_α_ distances between selected Ile residues. The differences in FOXO-DBDs core packing were further corroborated by calculating the contact area of residues from helices H1, H2 and H3 using the program AREAIMOL [48]. These calculations revealed the contact area of 893 Å^2^, 900 Å^2^ and 929 Å^2^ for FOXO1-, FOXO3- and FOXO4-DBD, respectively. In addition, all three apo forms also show different interactions between hydrophobic residues located on the interface between the N-terminal segment (Trp157 and Tyr162 in FOXO1), the H2–H3 loop (Tyr193 and Phe194), and the helix H3 (Trp206) (Figure 4). This cluster of hydrophobic residues forms part of the DNA-binding surface that makes contacts with the DNA backbone (Figure 4D). Furthermore, interactions in this region fine-tune the Forkhead domain DNA-binding specificity and affinity, most likely by affecting the position of the recognition helix H3 with respect to the helices H1 and H2, as shown for hepatocyte nuclear factor 3 (HNF-3, also known as FOXA), FOXC1 (FREAC3) and FOXD1 (FREAC4) proteins [13,49]. In the FOXO1-DBD structure, the side-chains of Tyr193, Phe194 and Trp206 interact in a face-to-edge fashion. In contrast, in FOXO3-DBD, the side-chain of Trp206 is rotated towards the side-chain of Tyr162 from the N-terminus of the helix H1 and makes a hydrogen bond with the hydroxyl group of Tyr193 from the helix H4. Interactions in FOXO4-DBD resemble those observed in FOXO1-DBD, but the side-chains of Phe138 and Trp150 interact in a face-to-face manner.

**Figure 5:**
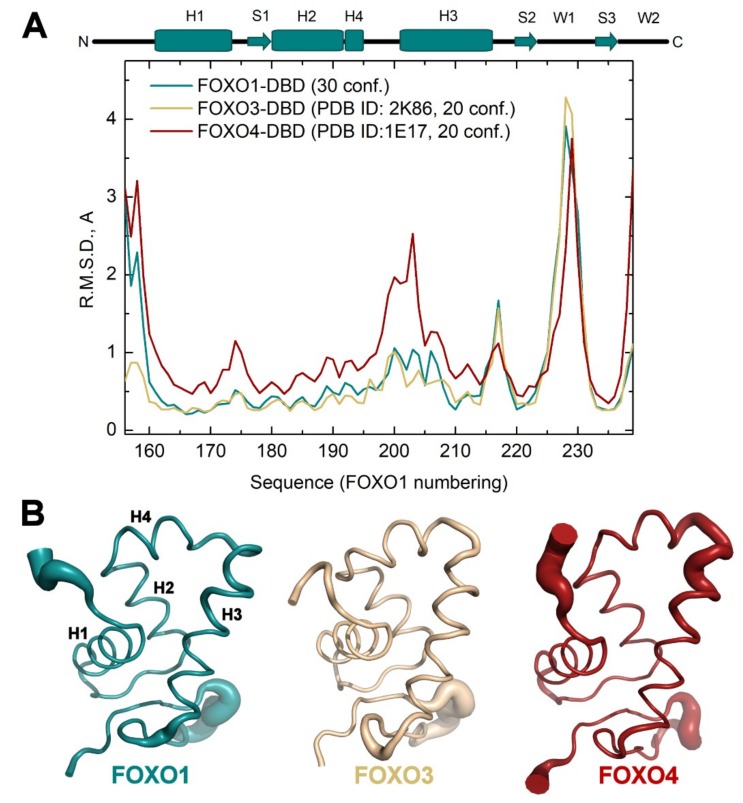
Conformational flexibility of FOXO-DBDs. (**A**) Root Mean Squared Deviation (R.M.S.D.) difference from average as a function of the sequence of FOXO1-DBD, FOXO3-DBD (PDB ID: 2K86 [46]) and FOXO4-DBD (PDB ID: 1E17 [45]). RMSD profiles were calculated using the MOBI server [50]. Secondary-structure elements (based on the FOXO1-DBD) are indicated at the top. (**B**) Sausage representation of NMR ensembles of FOXO1-DBD, FOXO3-DBD and FOXO4-DBD. The backbone diameter indicates the conformational mobility.

The comparison between the conformational flexibility of NMR solution structure ensembles using the MOBI server [50] revealed that FOXO1-DBD and FOXO3-DBD are comparable in this regard and show high flexibility (R.M.S.D. difference from average for C_α_ atoms > 1 Å) only within the H3-S2 and S2-S3 loops (Figure 5). Conversely, FOXO4-DBD exhibits a substantially increased conformational heterogeneity also in the H1-S1 loop and, especially, within the H4-H3 loop and N-terminal part of the helix H3.

Thus, the comparison of solution structures of apo FOXO-DBDs reveals differences both in their conformation and local flexibility. These differences presumably reflect variations in FOXO-DBD protein sequences, which are present, for example, within the α-helix H1 and C-termini of the helix H2 and β-strand S3 (Figure 2C and Figure S4). Accordingly, these differences may account for the distinct functions of FOXO proteins. For example, FOXO3- and FOXO4-DBDs have been shown to interact with the DNA-binding domain of transcription factor p53 [46,51]. Because the N-terminal segment of FOXO-DBD including aromatic residues W101 and Y106 (in FOXO4 numbering) is involved in this interaction, it is entirely possible that FOXO variants interact with p53 with different affinities due to the structural differences within the N-terminal parts of their DBDs. Moreover, the observed structural differences also suggest that FOXO-DBDs, although highly homologous, may differ in their DNA-binding affinities for different sequences.

### 3.3. Structural Differences Between Apo and DNA-bound Forms of FOXO1-DBD

**Figure 6:**
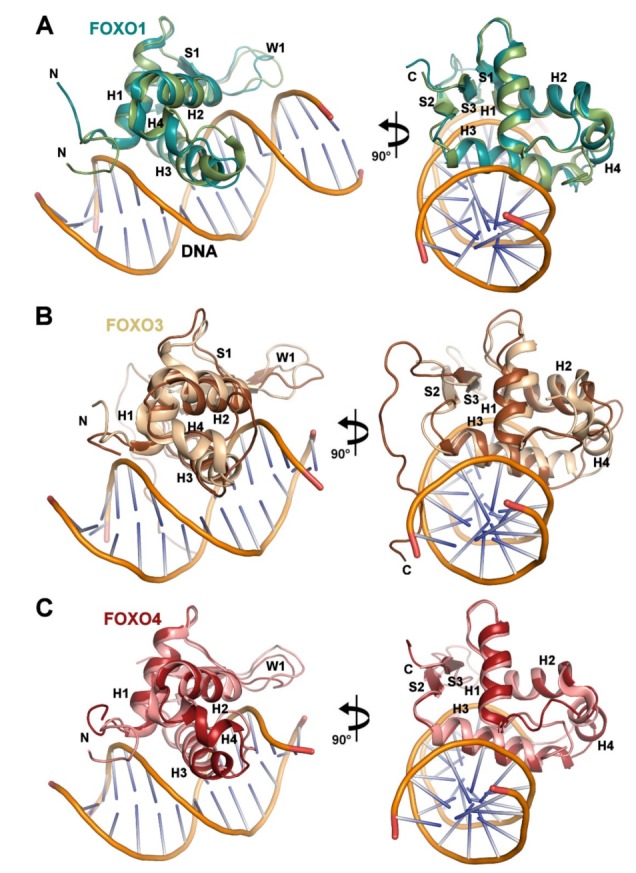
Comparison between apo and DNA-bound forms of FOXO-DBDs. (**A**) Superimposition of the solution structure of FOXO1-DBD (conf. no. 16, shown in dark cyan) with the crystal structure of the FOXO1-DBD:DNA complex (PDB ID: 3COA [7], shown in green). (**B**) Superimposition of the solution structure of FOXO3-DBD (conf. no. 11, PDB ID: 2K86 [46], shown in sand) with the crystal structure of the FOXO3-DBD:DNA complex (PDB ID: 2UZK [6], shown in brown). (**C**) Superimposition of the solution structure of FOXO4-DBD (conf. no. 3, PDB ID: 1E17 [45], shown in dark red) with the crystal structure of the FOXO4-DBD:DNA complex (PDB ID: 3L2C [8], shown in pink).

The structure of apo FOXO1-DBD can be superimposed with the crystal structure of the FOXO1-DBD:DNA complex (PDB ID: 3COA [7]) with an R.M.S.D. of 2.36 Å over 83 C_α_ atoms. Substantial differences are observed in the N-terminal segment, N-terminal part of the recognition helix H3, which is in the DNA-bound form shifted by 2 Å towards the N-terminal segment, loop between β-strands S2 and S3 (called wing W1), loop between H3 and S2, and loop between α-helices H2 and H3, that is, mainly in regions involved in DNA binding because the DNA-binding surface is formed by the helix H3, S2-S3 loop (wing W1) and N-terminal segment (Figure 6 and Figure S5) [7]. The positions of the α-helices H1 and H2 and β-strands S1-S3 remain virtually unchanged. Similar DNA-binding-induced conformational changes were observed in FOXO3-DBD and FOXO4-DBD [7,8]. The superimposition of apo and DNA-bound forms of FOXO3-DBD (2.72 Å over 81 C_α_ atoms) revealed additional changes in the position of the α-helix H1, whose C-terminal end is in the DNA-bound form, in comparison with the apo structure, which is shifted by 1.6 Å towards the β-strand S3 (Figure 6B) [6,46]. In addition, the C-terminus of H2, the H2–H3 loop and the N-terminus of H3 have substantially different conformations in both structures, but this is, at least partly, caused by crystal contacts because the H2–H3 loop of the FOXO3-DBD:DNA complex interacts with the DNA backbone of the symmetry-related copy of the complex. In FOXO4-DBD, the apo and the DNA-bound forms can be superimposed with an R.M.S.D. of 2.48 Å over 85 C_α_ atoms, and DNA binding has no effect on the positions of the three major helices H1, H2 and H3 (Figure 6C) [8,45]. Nevertheless, the recognition helix H3 of the FOXO4-DBD:DNA complex is compared to the apo structure shortened at the N-terminus by approximately one turn and thus similarly to the FOXO3-DBD:DNA structure. Although the interactions between hydrophobic residues at the interface of the N-terminal segment, H2-H3 loop, and helix H3 differ among apo FOXO-DBDs (Figure 4), the DNA-bound forms have similar arrangements of these residues (Figure 4D). This suggests that individual FOXO-DBDs undergo different conformational transitions upon their binding to the target DNA. This transition has been previously suggested by detailed analysis of interaction between FOXO4-DBD and DNA, which revealed that FOXO4-DBD binding to DNA involves a profound reduction of segmental dynamics of DBD upon complex formation [52].

## 4. Conclusions

FOX proteins are found in all eukaryotic species where they play a central role in cellular proliferation, differentiation, tumorigenesis and longevity (reviewed in [53,54]). The FOX family comprises more than 100 members classified into 17 subclasses designated A–Q. The members of the FOXO subclass (FOXO1, FOXO3, FOXO4 and FOXO6) are involved in a wide range of key biological processes including apoptosis, cell cycle, stress resistance, tumor resistance, differentiation, and metabolism (reviewed in [15,16,17]). Whereas FOXO1, FOXO3 and FOXO4 are widely expressed, FOXO6 is expressed mainly in neural tissues and developing brain [55]. Previous studies have shown that FOXO1 regulates the transcription of numerous genes involved in anti-oxidative stress, cell cycle arrest, apoptosis, autophagy, and metabolic regulation under both physiological and pathophysiological conditions [56,57]. Several studies have also shown that FOXO1 is a key regulator of hepatic glucose production and lipid metabolism because the liver-specific deletion of FOXO1 increases insulin sensitivity, fasting hypoglycemia and lipogenesis, whereas constitutively active FOXO1 blocks the insulin-mediated reduction of hepatic glucose production [58,59,60,61]. Therefore, FOXO1 is considered as a promising therapeutic target for diabetic hyperglycemia. In addition, FOXO proteins (FOXO1, FOXO3 and FOXO4) are putative targets for prevention cancer therapy-related drug resistance. However, the development of specific FOXO inhibitors requires a detailed understanding of structural differences between individual FOXO-DBDs. The high-resolution structure of FOXO1-DBD and its comparison with structures of other FOXO proteins revealed differences in both their conformation and flexibility that may account for the distinct functions of FOXO proteins. In addition, the availability of the resonance assignment for DBD of all three major FOXO types will enable us to use solution NMR spectroscopy to easily screen and characterize FOXO-small molecule ligand complexes at the atomic level and to study the selectivity of prepared compounds for various FOXO proteins.

## Figures and Tables

**Table 1:** Statistics for the final water-refined sets of structures.

Non-Redundant Distance and Angle Constrains	
Total number of NOE constraints	1712
Short-range NOEs	
Intra-residue (i = j)	445
Sequential (|i − j| = 1)	402
Medium-range NOEs (1 < | i − j | < 5)	325
Long-range NOEs (|i − j| ≥ 5)	530
Torsion angles	128
Hydrogen bond restrains	-
Total number of restricting constraints	1840
Total restricting constraints per restrained residue	16.3
**Residual constraint violations**	
Distance violations per structure	
0.1–0.2 Å	7.44
0.2–0.5 Å	2.46
> 0.5 Å	0
Root mean square (r.m.s.) of distance violation per constraint	0.02 Å
Maximum distance violation	0.48 Å
Dihedral angle viol. per structure	
1–10°	3.12
> 10°	0
r.m.s. of dihedral violations per constraint	0.55°
Maximum dihedral angle viol.	5.0°
**Ramachandran plot summary**	
Most favored regions	98.3%
Additionally allowed regions	1.7%
Generously allowed regions	0.0%
Disallowed regions	0.0%
**r.m.s.d. to the mean structure**	all/ordered ^1^
All backbone atoms	10.0/0.4 Å
All heavy atoms	10.1/0.8 Å
**PDB entry**	6QVW
**BMRB accession code**	34364

^1^ Residues with sum of phi and psi order parameters > 1.8.

## Supplementary Materials

The following are available online at https://www.mdpi.com/2073-4409/8/9/966/s1, Figure S1: ^1^H-^15^N HSQC spectrum of ^15^N-labeled mouse FOXO1-DBD (residues 156-269), Figure S2: Difference residue-residue (RR) distance maps, Figure S3: Comparison of mutual positions of the α-helices H1, H2 and H3, Figure S4: Position of non-conserved residues in FOXO-DBD sequences, Figure S5: Comparison of apo FOXO1-DBD with the structure of the FOXO1-DBD:DNA complex, Table S1: Root-mean-square deviations (R.M.S.D.) for the superimposition of apo FOXO-DBD structures.
