# Supplementary material for: Forkhead Domains of FOXO Transcription Factors Differ in both Overall Conformation and Dynamics
Source: Cells. 2019 Aug 24;8(9):966. doi: 10.3390/cells8090966 (PMC6770010; doi:10.3390/cells8090966)
Supplement: Supplementary file 1 [file cells-08-00966-s001.pdf]

Supplementary Materials

Table S1: Root-mean-square deviations (R.M.S.D.) for the superimposition of apo FOXO-DBD structures

	FOXO3-DBD	FOXO4-DBD
FOXO1-DBD	2.05[1] (1.47)[2]	2.12 (1.14)
FOXO3-DBD	-	2.50 (1.69)
FOXO4-DBD	2.50 (1.69)	-

[1]FOXO1-DBD (conf. #16, residues 156-239), FOXO3-DBD (conf. #11, residues 156-239), FOXO4-DBD (conf. #3, residues 96-179) were superimposed using all C_α atoms. R.M.S.D. values are expressed as Å.

[2]R.M.S.D. values assessed by superimposing FOXO-DBDs using only C_α atoms of three major helices H1, H2 and H3 (35 C_α atoms in total).

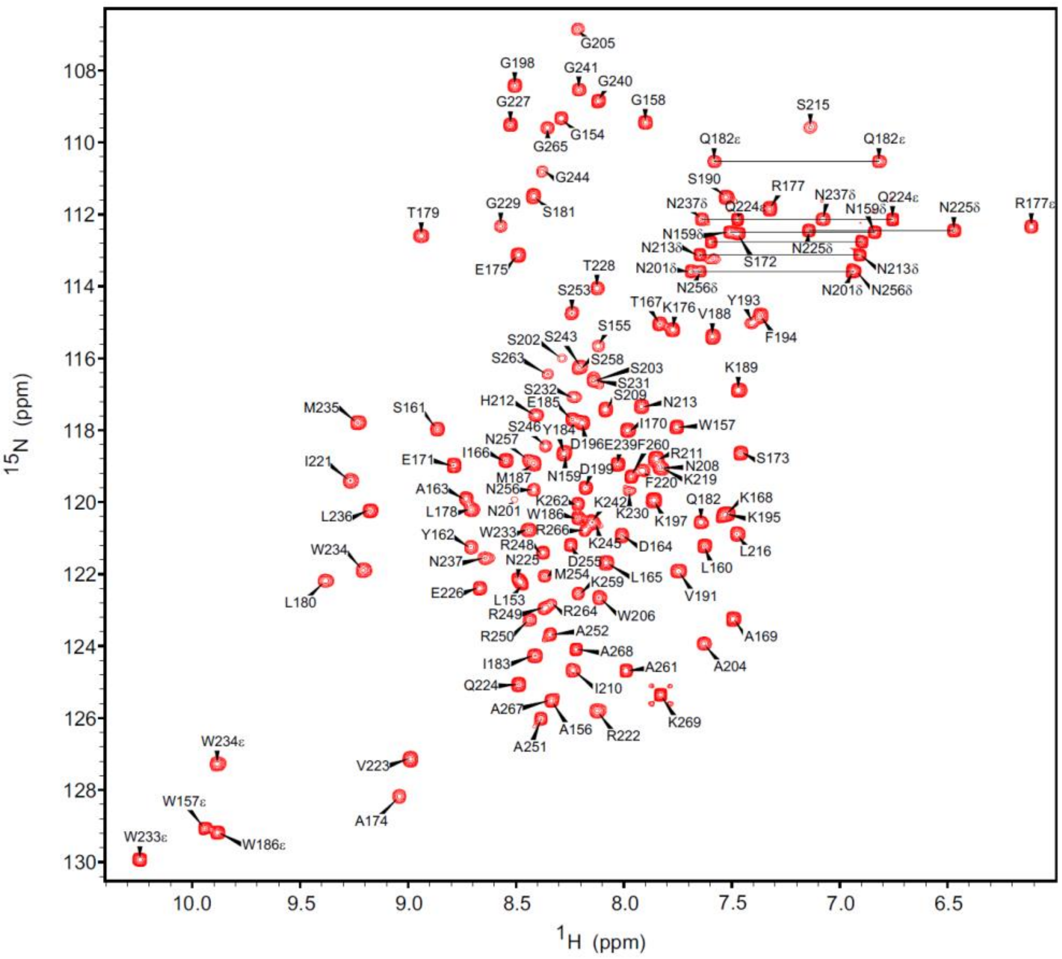

Figure S1. **^{1}H-^{15}N HSQC spectrum of ^{15}N-labeled mouse FOXO1-DBD (residues 156-269).** Complete resonance assignments were obtained for 104 of the 114 residues of FOXO1-DBD (91% of the molecule).

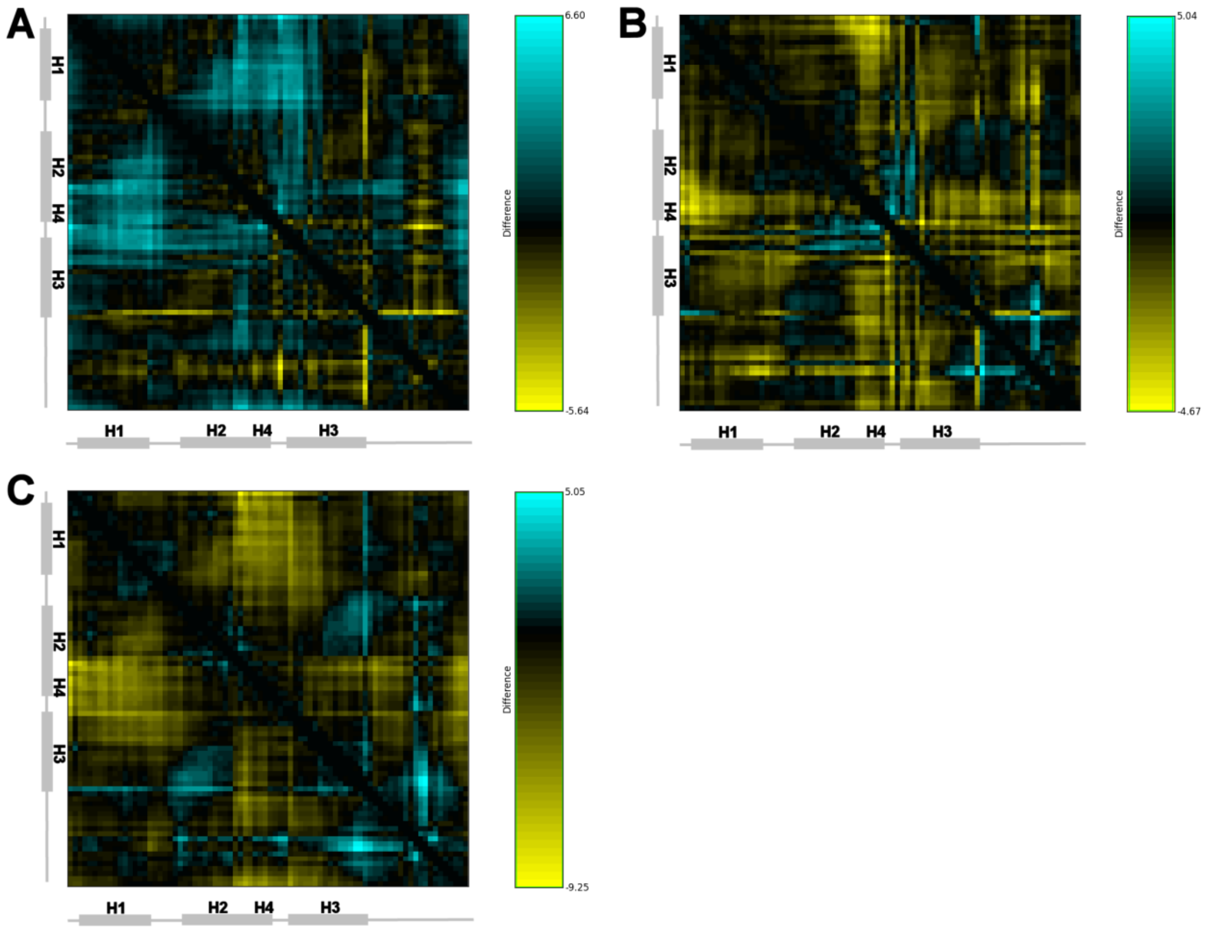

Figure S2. **Difference residue-residue (RR) distance maps.** Difference RR distance maps for the FOXO1-DBD/FOXO3-DBD (A), FOXO1-DBD/FOXO4-DBD (B) and FOXO3-DBD/FOXO4-DBD (C) pairs. Maps were calculated using the tool implemented in Chimera v1.11.2 [1]. Blue, yellow and black colors represent positive, negative and zero difference, respectively. The position of four helices H1-H4 is indicated by grey rectangles.

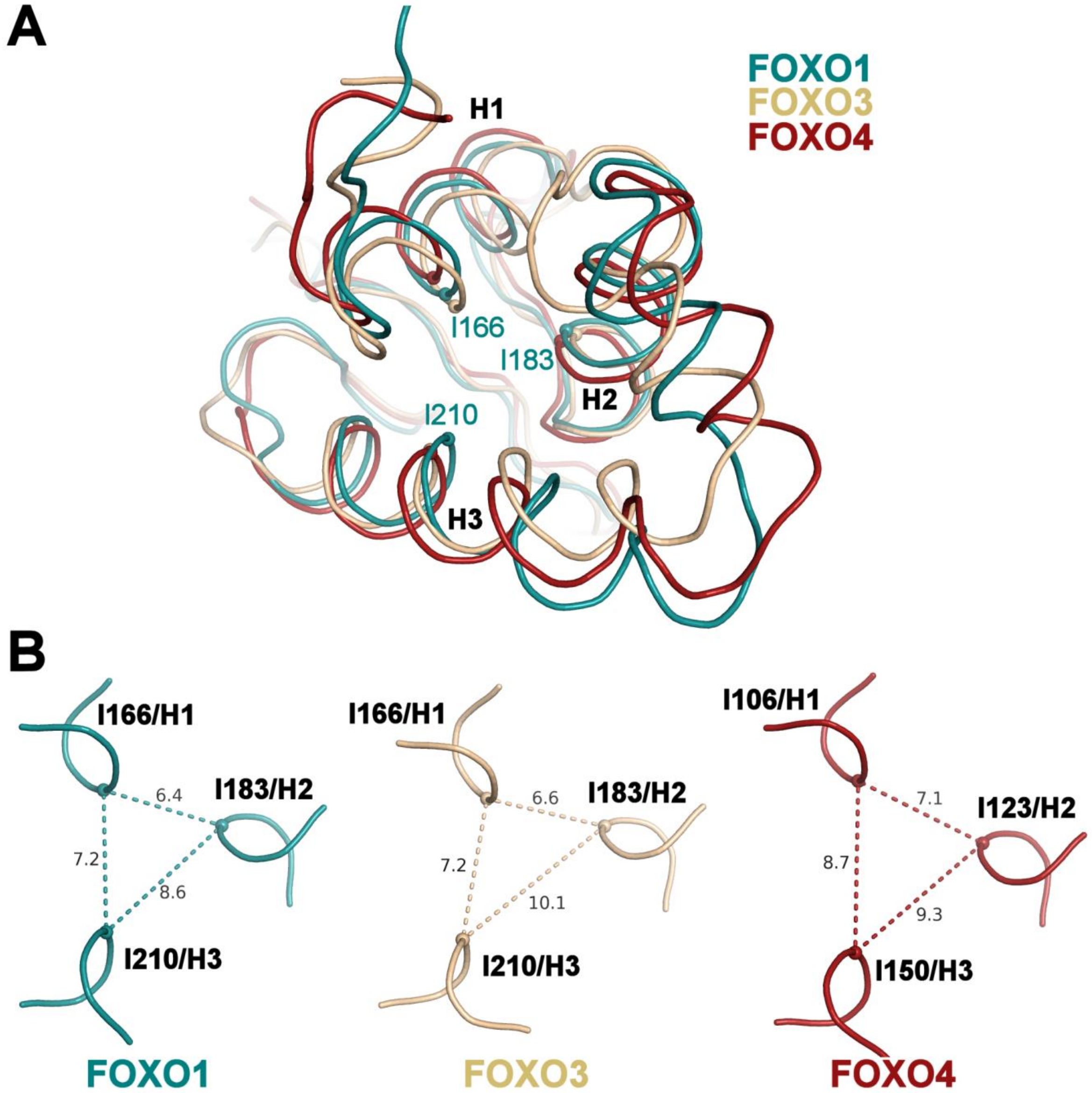

Figure S3. **Comparison of mutual positions of the α-helices H1, H2 and H3.** (A) Three Ile residues located approximately in the middle of the helices H1, H2 and H3 were selected (I166, I183 and I210 in the case of FOXO1). Positions of C_α atoms of these Ile residues are indicated by spheres. (B) Distances of C_α atoms of selected Ile residues from H1, H2 and H3 of FOXO1-DBD (conf. no. 16), FOXO3-DBD (conf. no. 11, PDB ID: 2K86 [2]) and FOXO4-DBD (conf. no. 3, PDB ID: 1E17 [3]).

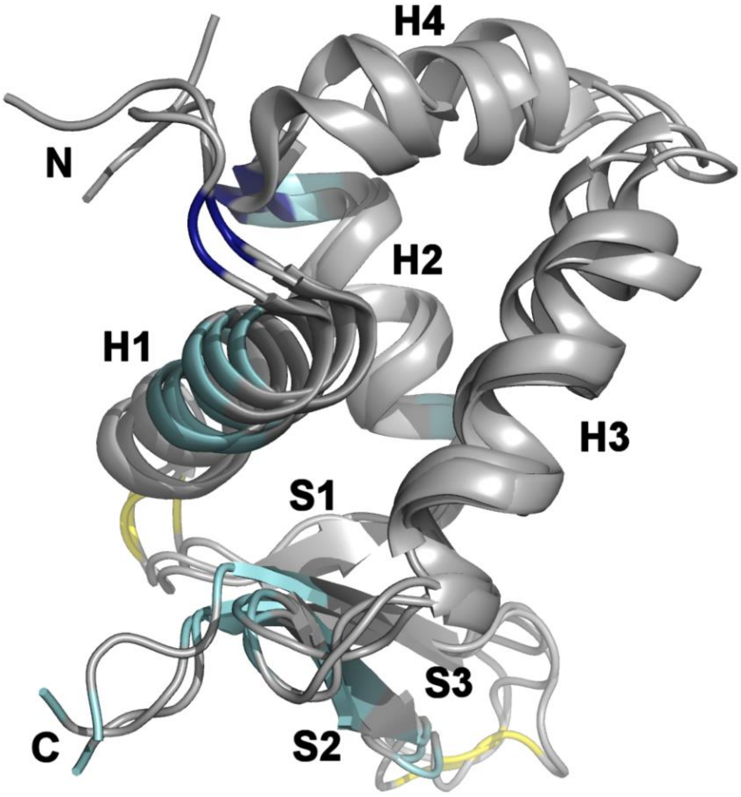

Figure S4. **Position of non-conserved residues in FOXO-DBD sequences.** Superimposition of representative conformers of FOXO1-DBD (conf. no. 16), FOXO3-DBD (conf. no. 11, PDB ID: 2K86 [2]) and FOXO4-DBD (conf. no. 3, PDB ID: 1E17 [3]). Colored regions indicate positions of non-conserved residues, non-conservative replacements are shown in dark blue, semi-conservative replacements are shown in cyan, and conservative replacements are shown in yellow.

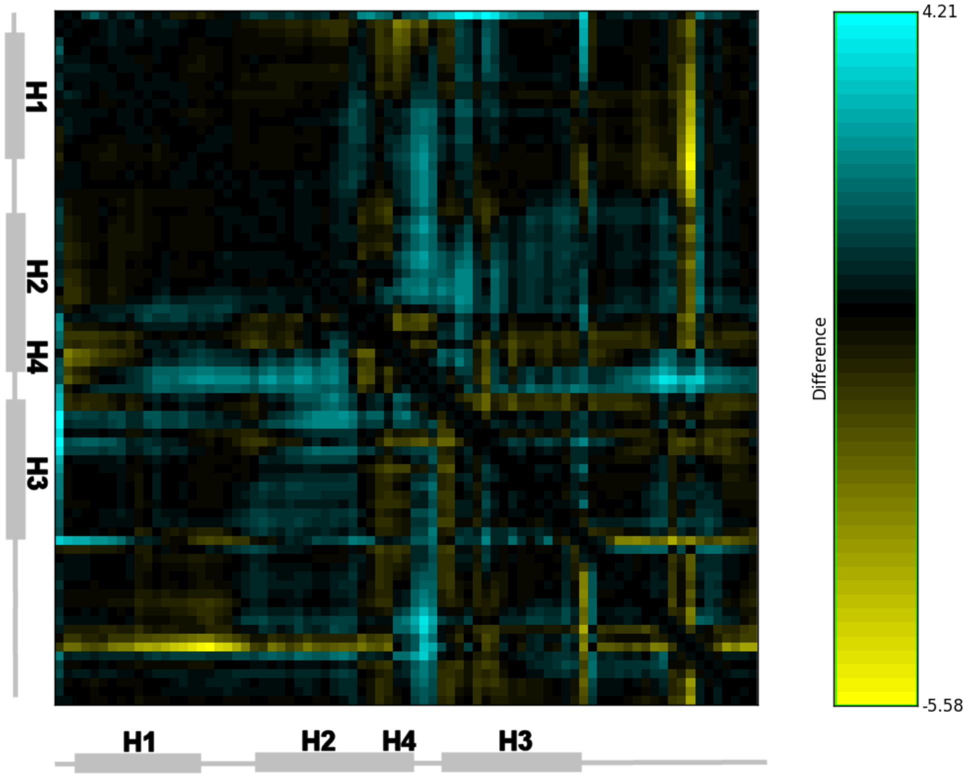

Figure S5. **Comparison of apo FOXO1-DBD with the structure of the FOXO1-DBD:DNA complex.** Difference residue-residue (RR) distance map for apo FOXO1-DBD and the structure of the FOXO1-DBD:DNA complex (PDB ID: 3COA [4]). The position of four helices H1-H4 is indicated in gray.

References

1. Chen, J.E.; Huang, C.C.; Ferrin, T.E. RRDistMaps: a UCSF Chimera tool for viewing and comparing protein distance maps. *Bioinformatics* **2015**, *31*, 1484-1486.
2. Wang, F.; Marshall, C.B.; Yamamoto, K.; Li, G.Y.; Plevin, M.J.; You, H.; Mak, T.W.; Ikura, M. Biochemical and structural characterization of an intramolecular interaction in FOXO3a and its binding with p53. *J Mol Biol* **2008**, *384*, 590-603.
3. Weigelt, J.; Climent, I.; Dahlman-Wright, K.; Wikstrom, M. Solution structure of the DNA binding domain of the human forkhead transcription factor AFX (FOXO4). *Biochemistry* **2001**, *40*, 5861-5869.
4. Brent, M.M.; Anand, R.; Marmorstein, R. Structural basis for DNA recognition by FoxO1 and its regulation by posttranslational modification. *Structure* **2008**, *16*, 1407-1416.
